# Antibody Response to Shiga Toxins in Argentinean Children with Enteropathic Hemolytic Uremic Syndrome at Acute and Long-Term Follow-Up Periods

**DOI:** 10.1371/journal.pone.0019136

**Published:** 2011-04-29

**Authors:** Romina J. Fernández-Brando, Leticia V. Bentancor, María Pilar Mejías, María Victoria Ramos, Andrea Exeni, Claudia Exeni, María del Carmen Laso, Ramón Exeni, Martín A. Isturiz, Marina S. Palermo

**Affiliations:** 1 División Inmunología, Instituto de Investigaciones Hematológicas, Academia Nacional de Medicina, Ciudad de Buenos Aires, Argentina; 2 Instituto de Leucemia Experimental (CONICET), Buenos Aires, Argentina; 3 Servicio de Nefrología, Hospital Austral, Pilar, Provincia de Buenos Aires, Argentina; 4 Departamento de Nefrología, Hospital Municipal del Niño, San Justo, La Matanza, Provincia de Buenos Aires, Argentina; Federal University of São Paulo, Brazil

## Abstract

Shiga toxin (Stx)-producing *Escherichia coli* (STEC) infection is associated with a broad spectrum of clinical manifestations that include diarrhea, hemorrhagic colitis, and hemolytic uremic syndrome (HUS). Systemic Stx toxemia is considered to be central to the genesis of HUS. Distinct methods have been used to evaluate anti-Stx response for immunodiagnostic or epidemiological analysis of HUS cases. The development of enzyme-linked immunosorbent assay (ELISA) and western blot (WB) assay to detect the presence of specific antibodies to Stx has introduced important advantages for serodiagnosis of HUS. However, application of these methods for seroepidemiological studies in Argentina has been limited. The aim of this work was to develop an ELISA to detect antibodies against the B subunit of Stx2, and a WB to evaluate antibodies against both subunits of Stx2 and Stx1, in order to analyze the pertinence and effectiveness of these techniques in the Argentinean population. We studied 72 normal healthy children (NHC) and 105 HUS patients of the urban pediatric population from the surrounding area of Buenos Aires city. Using the WB method we detected 67% of plasma from NHC reactive for Stx2, but only 8% for Stx1. These results are in agreement with the broad circulation of Stx2-expressing STEC in Argentina and the endemic behavior of HUS in this country. Moreover, the simultaneous evaluation by the two methods allowed us to differentiate acute HUS patients from NHC with a great specificity and accuracy, in order to confirm the HUS etiology when pathogenic bacteria were not isolated from stools.

## Introduction

Verocytotoxin-producing *Escherichia coli* (*E. coli*), also referred to as Shiga toxin (Stx) -producing *E. coli* (STEC), infection is associated with a spectrum of clinical manifestations that include diarrhea, hemorrhagic colitis, and hemolytic uremic syndrome (HUS) [1]–[3]. Systemic Stx toxemia is considered to be central to the genesis of HUS [2] because there is cumulative evidence demonstrating systemic Stx-mediated damage to vascular endothelial cells in the kidney, gastrointestinal tract, and other organs and tissues [4].

Stxs are a family of protein toxins that share a structure of polypeptide subunits consisting of an enzymatically active A subunit (approx 32 kDa) that is linked to a pentamer of B (binding) subunits (approx 7,5 kDa) [5]. The holotoxin binds to the glycosphingolipid receptors, preferentially globotriaosylceramide (Gb3), on the surface of eukaryotic target cells and it is internalized by receptor-mediated endocytosis [6]. The A subunit is proteolitically nicked to an active A1 fragment (aprox 27.5 kDa) that acts on the 28S ribosomal subunit to inhibit protein synthesis [7]. Among the Stxs produced by human STEC isolates, Stx2 and Stx2c show the highest association with HUS [8]. Stx1 is serologically distinct from Stx2 (and Stx2c) and these toxins do not show cross-neutralization by homologous antisera in Vero cell monolayers [7], [9]. On the other hand, Stx2 is completely neutralized by anti-Stx2c antiserum, whereas Stx2c is only partially neutralized by Stx2 antiserum [10].

Laboratory diagnosis of STEC O157 infections relies on the pathogen isolation from stools [8], detection of Stx in the fecal filtrates [11], and/or anti-Stx serum antibodies [12]. Although some reports have shown that patients develop rising levels of Stx antibodies following STEC infection [13]–[15], little is known about the nature and duration of the serum anti-toxin response and the role of these antibodies in immunity. The earliest method used to test the presence of anti-Stx-antibodies has been the standard neutralization assay (Stx-Nab), which is tedious and difficult to standardize. In addition, Stx2-Nab assay has been shown to detect nonspecific neutralizing activity in serum associated to a component of the serum high-density lipoprotein fraction, rather than specific antibodies [16]. Some progress has been made through the development of enzyme-linked immunosorbent assays (ELISA) [13] and western blot [16], [17]. However, the diagnosis of HUS in Argentina is mainly based on clinical parameters, and specific microbiological studies are only done by the National Reference Laboratory from the National Health Surveillance System [18]. Then, the application of those immunoassays to detect the presence of specific antibodies to Stx2 for serodiagnosis and seroepidemiological studies has been very limited but it can be improved and generalized if simple and inexpensive techniques are standardized, and show applicability and pertinence in our country.

Measurement of antibodies to O157 lipolysaccharide has been widely used for serological diagnosis of HUS associated to *E. coli* O157:H7 infection [18]–[20], because O157:H7 is epidemiologically the most frequent seropathotype associated to HUS. However, the improvement of microbiological detection methods has reported an increasing frequency of HUS cases associated to non-O157 serotypes, such as O26:H11, O103:H2, O111:NM, O121:H19, and O145:NM [21], [22].

An increasing frequency of anti-Stx antibodies has been reported in higher-age population which is in general refractory to HUS [23]. In addition, anti-Stx2 seroreactivity has been correlated with the absence of symptoms in family outbreaks of STEC infection [24], [25]. This evidence together with the almost null recurrence of the enteropathic form of this disease, suggest that HUS resistance may be associated with increasing immunity, possibly to Stx2.

The objectives of the present study were 1) to develop a standard antibody ELISA to detect anti-Stx2 B subunit, and a WB assay against the whole Stx2 and Stx1 proteins; 2) to correlate the results from anti-Stx2B ELISA with those from anti-Stx2 WB and to validate them to be used in our population; and 3) to study the presence of antibodies against Stx2 A and B subunits in normal healthy children and HUS patients from Argentina.

## Materials and Methods

### Ethics statement

The study was approved by the Ethical Committee of Children's Hospital, San Justo, Provincia de Buenos Aires. All HUS patients and controls were enrolled in this study between March 2003 and September 2008 after the written informed consent from their parents had been obtained.

### Patients and controls

All children were from the same urban area. The criteria for HUS diagnosis were microangiopathic hemolytic anemia with schizocytes, thrombocytopenia (platelet count <150×10^9^/L) and acute renal failure (serum creatinine level higher than the normal values considering the age of the patient). All patients developed HUS after gastroenteritis consisting of bloody diarrhea (n = 56). Sixty percent of the children were positive for STEC (Stx2 and/or Stx2c positive), diagnosed by stool culture. Forty seven percent were boys and 53% were girls, age range 8 to 108 months; median, 32 months; mean, 40 months (standard deviation {SD} 26 months). Blood samples (2 ml) were obtained for biochemical studies by venopuncture into EDTA plastic tubes at hospitalization. Plasma samples were immediately separated and stored at −20°C until use.

Plasma samples collected after different times during HUS follow-up (from 18 days to 213 months after the onset of HUS; median = 51 months; mean = 65 months; 1SD = 60 months) were available from 49 patients (37% male, 63% female). Since HUS-recovered patients (HUSrec) should be followed-up for nephrological controls at least up to adolescence, the samples from HUSrec patients were obtained when they assisted to the out-patient nephrology department after a non-standard period of time since the acute disease.

Blood samples from normal healthy children (NHC, n = 72) admitted for routine surgical procedures were collected and processed identically (age range, 2 to 156 months; median = 60 months; mean = 59 months; 1SD = 50 months). They had no history of diarrhea for the preceding 6 months and they were not associated with an outbreak or sporadic case of HUS.

### Shiga toxin preparation and purification

The complete gene for Stx2 was amplified by PCR from total DNA from *E.coli* O157:H7 C600 (933W) by using the primers previously reported [26]. The complete gene for Stx1 was amplified by PCR from total DNA from the same strain *E.coli* O157:H7 C600 (933W) with primers upstream (5′ GAT ATG TTA AGG TTG CAG CTC TC-3′) and downstream (5′ GGG CTA TTC TGA GTC AAC GG-3′). The resulting fragments (1,422 bp for Stx2 and 1,396 bp for Stx1), which included the original promoter region, were cloned in pGEMT Easy vector (Promega, Madison, WI), thus generating the plasmids pGEMTStx2 and pGEMTStx1. The culture of recombinant *E. coli* JM109 strain transformed by the recombinant plasmids containing the Stx1 or Stx2 sequences, was obtained by incubation overnight at 37°C with shaking at 200 rpm in Luria-Bertani (LB) broth (Difco Laboratories, MD) supplemented with 100 µg/ml ampicilin (Sigma-Aldrich, St. Louis, MO). Bacterial cells were centrifuged and the resultant pellet was resuspended in PBS containing 1 mM phenylmethylsulfonyl fluoride (PMSF) protease inhibitor (Gibco, Grand Island, NY) (PBS-PMSF) and lysed by sonication. After centrifugation (14,000 rpm, 20 min 4°C) the supernatant from JM109/pStx2 was precipitated with amonium sulfate solution (75%), purified as previously described [17] and stored at −20°C until its usage.

Stx1 was purified by affinity chromatography with commercially available Globotriose Fractogel (IsoSep AB, Tullinge, Sweden) as previously described [27]. Briefly, 1 ml of the supernatant from JM109/pStx1 was applied to 1 ml Globotriose Fractogel column. The column was incubated at 4°C for 2 h and washed three times with 5 ml of PBS. Stx1 was eluted from the column with 5 ml of PBS containing 4 M MgCl2. The eluted material was pooled, dialyzed with PBS, and concentrated by centrifugation filtration using the Ultra system (Millipore, Bedford, MA) up to a concentration of 200 µg protein/ml. The concentrated toxin preparation was examined for homogeneity by sodium dodecyl sulphate-polyacrylamide gel electrophoresis (SDS-PAGE).

### Western blot assay to detect anti-Stx 2 and –Stx 1 IgG (H+L)

This assay was adapted from the method reported by Karmali et al. [23]. Briefly, a standard concentration of purified Stx2 or Stx1 (80 µg) was resolved into its A and B subunits by SDS-PAGE, by using 6% stacking and 17.5% separating gels and electroblotted onto polyvinylidene difluoride (PVDF) membranes (Bio-Rad Laboratories, Hercules, CA) overnight (20 v). Membranes were stained with Ponceau red to confirm protein transfer. After overnight blocking in Tris buffer (Tris 50 mmol/L; pH 7.4) containing 5% skim milk and 5% goat serum (Chemicon International, Billerica, MA), each membrane was cut into longitudinal strips. The strips were incubated overnight at 4°C in the plasma specimens diluted 1∶100 in Tris buffer containing 5% skim milk and 5% goat serum. After washing, the strips were incubated for 1 h at 37°C with 1∶10000 goat anti-human IgG (H+L) peroxidase conjugate (Chemicon International) in Tris buffer containing 3% skim milk and 3% goat serum. After three washes in Tris buffer, the strips were developed using a chemiluminiscent detection system (ECL; Amersham Pharmacia Biotech, United Kingdom). The strips were exposed to a Kodak AR film (Kodak, Rochester, NY) for 10 min, and the film was then developed. Each plasma sample (patient as well as control specimens) was tested at least twice. The results of the WB were read in a blinded fashion.

### Stx2 B subunit (Stx2B)

In order to obtain the Stx2 B subunit, the *stx2b* gene was amplified by PCR and cloned as previously described [28]. Briefly, the *stx2b* gene was cloned in the pQE-70 vector (QIAGEN, Germantown, MD) thus generating the plasmid pStx2B. Competent *E. coli* DH5α (Bethesda Research Laboratories, Carlsbad, CA) were transformed with the pStx2B, were grown in LB supplemented with 100 µg/ml ampicilin until an OD_600_ of 0.6 at 37°C with shaking (200 rpm), and then were induced with 1 mM isopropyl β-D-1-thiogalactopyranoside (IPTG) (Sigma-Aldrich) over 5 hours. The supernatant was filtered (0.22 µm) and treated with ammonium sulphate to 75% saturation. The precipitate was collected by centrifugation, then resuspended in PBS-PMSF, dialyzed overnight at 4°C and stored at −20°C until its usage.

### Detection of anti-Stx2B IgG by ELISA

This assay was adapted from the method previously reported [24] with some modifications. Briefly, 96-well microtiter plates (GBO, Germany) were coated by incubating overnight with 0.5 µg Stx2B per well in 15 mM carbonate, 25 mM bicarbonate (pH 9.6) at 4°C. As antigen controls, wells were incubated with 0.25 µg supernatant from DH5<alpha> (pQE-70), which lacks the B subunit sequence, or PBS, and they gave equivalent results. Then, 250 µl of PBS containing 1% BSA was added to each well and the plates were incubated for 2 h at 37°C. The plates were washed three times with PBS containing 0.1% Tween 20 and incubated for 2 h at 4°C with the plasma samples diluted 1/4000 in PBS containing 0.05% Tween 20 and 0.5% BSA. After three washes, the plates were incubated overnight with 1/4000 goat anti-human IgG horseradish peroxidase conjugated in PBS containing 0.05% Tween 20 and 0.5% BSA at 4°C. As control for the unspecific stuck of the secondary antibody, 100 µl of PBS was added instead of plasma samples. After three more washes, 100 µl of substrate solution (0.1 M citrate-phosphate pH 5, 1 mg/ml o-phenylenediamine dihydrochloride [OPD] (Sigma-Aldrich), 30% H_2_O_2_) was added to each well and incubated for 20 min at room temperature in darkness. The reaction was terminated by the addition of 50 µl of 4 N sulfuric acid and the optical density values at 492 nm (OD_492_) were measured in a microplate ELISA reader. To obtain the net ELISA value, each plasma specimen was tested in duplicate and the mean OD_492_ value was calculated subtracting the OD_492_ value of the test well from the OD_492_ value of the antigen control well. In order to determine the cut-off value for the ELISA test, we calculated the mean value + 2SD from those plasma samples from NHC group that did not show specific bands by WB studies (mean = 0.119, 2SD = 0.107, n = 18). Taking into account the high prevalence of Stx antibodies in the NHC, we also evaluated plasma samples from a small group of newborn children (n = 5) by ELISA test (OD_492_ mean = 0.116, 2SD = 0.087), and the mean value + 2SD for them was not different to that for NHC group. Thus, OD_492_ = 0.226 was considered as the cut-off for the ELISA test and absorbance values below this OD were considered as IgG-negative for Stx2.

### Statistical methods

Frequency values were compared with the Chi-square test for independence and trend (two by three tables) or Fisher's exact test (two by two table) as indicated. Median values were compared with Wilcoxon Signed Rank test as indicated.

## Results

### Antibodies against the A and/or B subunit of Stx2 and Stx1

**Figure 1:**
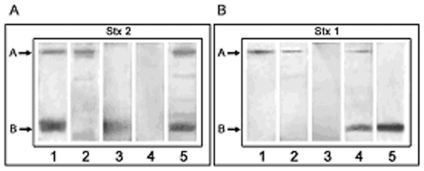
Antibodies against Stx2 and Stx1. Representative immunoblot strips from HUS or healthy children plasma reactive to Stx2 (A) and Stx1 (B) are shown. A) Lane 1 shows a mouse-polyclonal plasma reactive against Stx2 A and B subunits; positive human plasma showed reactivity to either the A subunit alone (lane 2), the B subunit alone (lane 3), or both subunits (lane 5). Samples that showed a complete absence of binding signal were considered negative (lane 4). B) Lane 1 shows a mouse-polyclonal plasma reactive against Stx1 A subunit, and lane 5 shows a monoclonal hybridoma reactive against Stx1-B subunit (13C4, ATCC CRL-1794). Positive human plasma showed Stx1-binding signal to either the A subunit alone (lane 2) or both subunits (lane 4). Samples that showed a complete absence of binding were considered negative (lane 3). Each plasma sample was assayed at least twice. When discrepancy between the results was observed, a third WB was made in order to rule out the wrong result.

Purified Stx2 and Stx1 were resolved by SDS-PAGE, after transferring it onto PVDF membranes, and two bands corresponding to the molecular mass of A and B subunits from each toxin were identified with either control mouse-polyclonal antibodies anti-Stx2 or anti-Stx1 (Fig. 1A and B). Human plasma that were positive for Stx2 (Fig. 1A) were reactive with either the A subunit alone, the B subunit alone, or both subunits. Although the intensity of the reactivity was variable, the strongest signals were detected in HUS or HUSrec plasma. Human plasma that were positive for Stx1 antibodies (Fig. 1B) were reactive with either the A subunit alone, or both subunits. At sample dilution assayed none of Stx2-positive plasma were positive for Stx1.

### Frequencies of Stx2 and Stx1 antibodies

**Figure 2:**
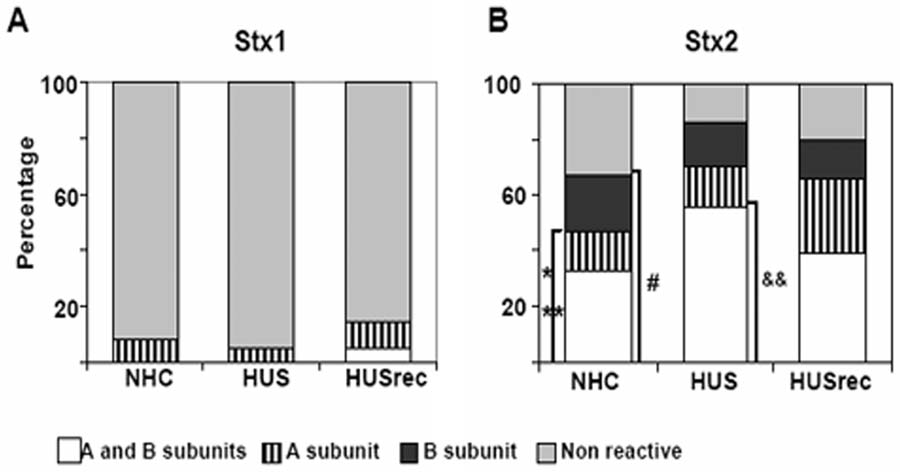
Frequencies of Stx2 and Stx1 antibodies. The frequency of reactive plasma samples against Stx1 (**A**) or Stx2 (**B**) in age-matched normal healthy children (NHC) (n = 72), in patients with enteropathic HUS at acute period (n = 56), or after recovery (HUSrec) (n = 49) are shown. Each bar represents non-reactive plasma samples (light grey) or samples reacting with only B subunit (black), only A subunit (striped), or with both subunits (A and B) (white) of Stx1/Stx2 assayed by WB analysis. **A**) Significant differences were not observed between the groups (p = 0.55, Chi-square test). **B**) ^#^p<0.05 vs HUS and HUSrec, Chi-square test; *p<0.05 vs HUS and HUSrec, ** p<0.01 vs NHC, Fisher's exact test.

Plasma samples reacting with no subunits or with the A and/or B subunit of Stx2 and/or Stx1 are shown in Fig. 2.

When samples were tested against Stx1, a similar low frequency of reactive samples was observed in all clinical groups (Fig. 2A) (HUS = 5%, HUSrec = 14%, NHC = 8%; p = 0.55, Chi-square test). All positive plasma samples were reactive only against Stx1-A subunit, except one that was reactive for both, A and B subunit of Stx1.

The overall frequency of reactive plasma with at least one subunit of Stx2 in NHC group (67%) was significantly lower than the frequency in HUS (86%) and HUSrec (82%) clinical groups (Fig. 2B) when compared by the Chi square test (p<0.05).

HUS patients developed antibodies against both subunits of Stx2 more often than NHC control group (HUS = 56% NHC = 33%; p<0.01, Fisher's exact test). However, this difference was lost during follow-up period (HUSrec = 39%; p = 0.39, Fisher's exact test) (Fig. 2B). Considering plasma samples reactive to the A subunit, alone or together with the B subunit, we found that HUS patients presented antibodies against the Stx2 A subunit significantly more often than controls did (HUS = 70%, NHC = 47%, p<0.01, Fisher exact test). This increase in the frequency of Stx2 A subunit antibodies was still evident during the follow-up period (HUSrec = 66%, p<0.05 vs NHC, Fisher's exact test). Acute HUS patients also showed a significantly higher frequency of antibodies against the B subunit than NHC patients (HUS = 72%, NHC = 53%, p<0.05, Fisher's exact test), but this difference was lost during follow-up (HUSrec = 53%) (Fig. 2 B).

### Antibodies against Stx2 B subunit measured by an ELISA assay

**Figure 3:**
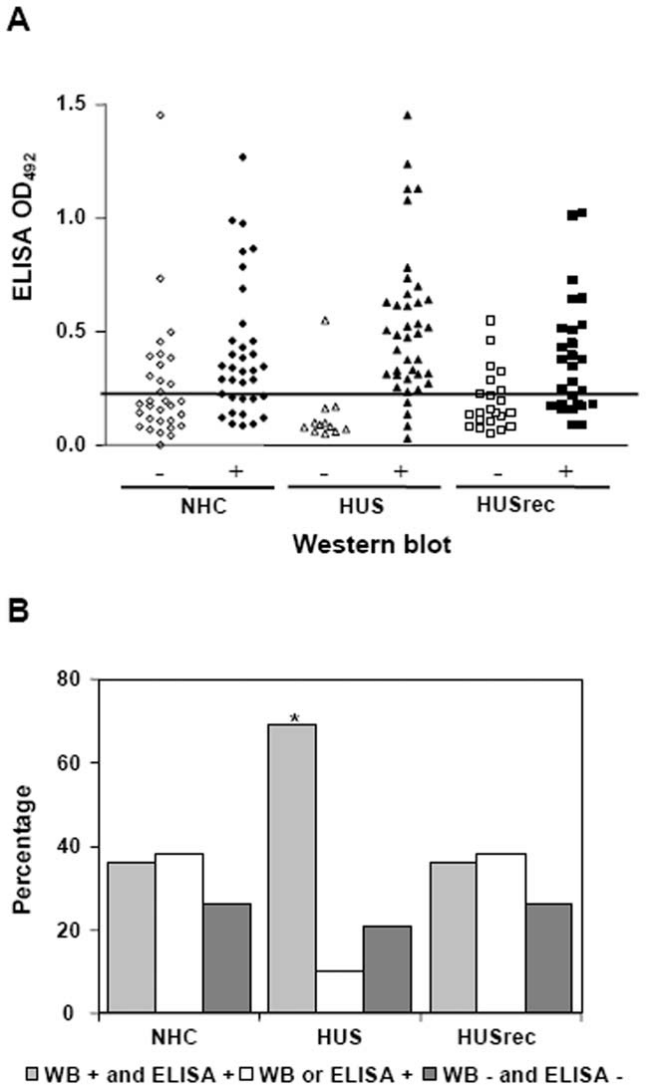
Antibodies against Stx2 B subunit measured by ELISA. **A**) Absorbance values by ELISA assay detecting anti-Stx2 B IgG in plasma from NHC, HUS or HUSrec groups were segregated according the result (positive or negative) obtained by WB for the Stx2 B subunit. OD_492_ values obtained are individually depicted. The horizontal whole line (OD_492_ = 0.226) indicates the cut-off value to consider a plasma sample as positive or negative. **B**) Relationship between results of anti-Stx2 B antibodies obtained by ELISA and WB assays. Bars represent the frequency of positive plasma against Stx2 B subunit by the two methods (light grey), positive sera by at least one of the methods (white) and negative by the two methods (dark grey) in normal healthy children (NHC), HUS patients at acute period (HUS) or after recovery (HUSrec). *p<0.0005 vs NHC and HUSrec, Chi-square test.

The anti-Stx2 B subunit antibody response was also examined by an ELISA test, by using purified Stx2 B subunit as the coating protein. OD_492_ values for each plasma sample from different clinical groups tested at 4,000-fold dilution are shown in Fig. 3A.

Absorbance values for each sample were plotted according to their WB result (segregated as positive or negative for B subunit), and into their corresponding clinical group (Fig. 3A). Although these results showed that WB-positive plasma samples from different clinical groups had a wide range of OD_492_ absorbance when assayed by ELISA, the median absorbance of positive samples of HUS group was higher than the NHC group (HUS = 0.51, n = 35; NHC = 0.40 n = 23; p<0.01, Wilcoxon Signed Rank test). However, it was not significantly different to the HUSrec group (HUSrec = 0.45, n = 17; p = 0.051, Wilcoxon Signed Rank test).

In addition, Fig. 3A shows that plasma from 35 out of 39 HUS patients presented a reactive band against Stx2 B subunit by WB and had an absorbance ELISA value over the cut-off (89.7%), while only 1 out of 12 HUS samples was non-reactive by the WB assay and had an absorbance over the cut-off value (8.3%), confirming the strength and specificity, respectively, of the combined test-assaying.

On the other hand, plasma from 17 out of 27 HUSrec children were reactive for Stx2 B subunit by WB and positive by ELISA assay (63.0%), and only 23 out of 35 NHC children showed positive results by both ELISA and WB assays (65.7%) (Fig. 3A). When WB-negative sera against the Stx2 B subunit were assayed by ELISA, we observed very low and similar OD_492_ values in the three clinical groups.

Finally, Fig. 3B shows that HUS group had the highest percentage of positive plasma samples by the two methods (p<0.0005 vs NHC and HUSrec, Chi-square test), suggesting that the combination of both techniques add strength to the result.

### Kinetics and duration of antibody response against Stx B subunit in HUS patients

**Figure 4:**
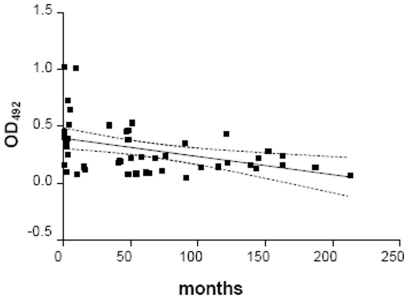
Correlation of anti-Stx2 B IgG ELISA plasma values from HUSrec patients with the time since the enteropathic HUS episode. OD_492_ values obtained at different times (months) since the HUS episode are individually depicted. The regression line ± 2SD is indicated by the whole and dashed lines, respectively. Correlation coefficient, r = −0.41, p<0.005.

Since HUS group showed higher frequency of positive samples for B subunit by WB (Fig. 2) and higher ELISA titers (Fig. 3A and 3B) than HUSrec group, we plotted ELISA titers over time (Fig. 4).

Although plasma samples from HUSrec group were not collected after a standard predetermined period of time after the onset of disease, it can be clearly stated that absorbance levels of anti-Stx2 B subunit IgG by ELISA were higher during the first year after illness, following a slow decrease over time. It is important to highlight that patients after more than 10 years of follow-up were also included in this study, and they still have reactivity against B and/or A subunit.

## Discussion

In spite of the high circulation of STEC strains in Argentina, in particular those producing Stx2 [8], local information about the frequencies of anti-Stx2/Stx1 antibodies in HUS cases and healthy children is very limited. This is because, at least in part, the evaluation of anti-Stx2/Stx1 antibodies is not routinely done by clinical laboratories. Although the presence of neutralizing activity in HUS patient's sera by using Vero cell monolayers is routinely performed by the National Reference Laboratory from the National Health Surveillance System, it is known that a very low neutralizing activity is generally observed and not in all patients [12] . For this reason, several authors have reported more sensitive tests such as ELISA [29] or immunoblotting assay [16], [23] for anti-Shiga toxin antibodies detection. Our work indicates that Western blotting and ELISA can be successfully used also in Argentina to detect antibodies to Stx2 in both healthy and HUS children. Since both methods combine different antigenic proteins: B subunit of Stx2 or the whole holotoxin, either native or denatured, they probably detect different antibodies thus enhancing the spectrum of antibody detection. It is interesting to highlight that the association between the positive results obtained by ELISA and WB techniques was stronger in HUS group than in NHC or HUSrec groups (Fig. 3B), probably as a consequence of higher antibody titers in plasma from HUS group than those in plasma from NHC or HUSrec groups. Moreover, the simultaneous evaluation by the two methods allowed us to differentiate HUS acute patients from normal healthy children with a great specificity and accuracy, in order to confirm the HUS etiology when non-pathogenic bacteria were isolated from stools.

Serological examination of sporadic HUS cases provide a useful tool for diagnosis of STEC infection wherein STEC isolates or free fecal Stx2 are not detected [30]. We found a very similar frequency of antibodies against Stx2 in HUS patients and HUSrec group (86% and 82%, respectively), even when several years had elapsed since their illness. In comparison, only 67% NHC patients showed anti Stx2-antibodies. Although this frequency was significantly lower compared to those in HUS patients, it is important to point out that it was significantly higher than the frequencies reported in healthy children from other countries such as Germany (10%) [17] or Canada (46%) [23] for an age-matched population. This result could be ascribed to the high circulation of Stx2-producing strains in Argentina [8], [31], and could be associated to the HUS endemic behavior in this country. The existence of a large non-symptomatic population of adults that carry the pathogenic strains could contribute to this behavior through a person-to-person transmission. Indeed, STEC-positive adults or children are often detected when a household child undergoes STEC-associated bloody diarrhea or HUS [32]. Moreover, 51% of HUS household contacts have been reported to have neutralizing anti-Stx2 antibodies in Argentina [25]. Reports demonstrating diarrheal illness in a family member as a risk factor for developing HUS in Canada also support person-to-person transmission [33]–[35].

In sharp contrast, the percentage of positive plasma for Stx1 was below 15% in both, NHC and HUS Argentinean groups. Moreover, these frequencies are similar to those found in Europe [36] and in the United States [13] comparing both, healthy and HUS populations. The much higher frequency of antibodies to Stx2 than to Stx1 in controls together with the observation of a high prevalence of Stx2/Stx2c-producing STEC in beef cattle [37], as well as in stools from post-enteric HUS cases in Argentina [8], [31], suggest that Stx2-producing strains are much more common than Stx1-producing ones.

Regarding the specificity of Stx2 antibodies, we observed that HUS patients (acute+HUSrec) showed higher frequencies of anti-Stx2 A subunit antibodies compared to controls, similarly to previous reported data in other countries [2], [25]. Moreover, an important finding of the present study was that HUS patients have a long lasting humoral immune response, particularly against the A subunit. This fact has several theoretical and practical implications. First of all, these results go against the hypothesis that Stx2 is cytotoxic for B lymphocytes, preventing the induction of anti-Stx2 antibody response, already previously questioned by Barrett et al. [13].

Secondly, because STEC/Stx2 is highly endemic in Argentina, it is possible that an asymptomatic or auto-limited gastrointestinal infection with pathogenic strains was insufficient to stimulate a detectable antibody response to Stx2 A subunit. On the other hand, whether the anti-Stx2 A subunit response observed in HUS children would result from a booster response during a second exposure, or as consequence of a high enough bloodstream concentration of Stx2, is a matter for discussion. Although we used anti-IgG as secondary antibody in this study, it reacted against the heavy- and light-chain, so the WB could detect not only IgG but also, potentially, IgM and IgA. It is likely, however, that among the patients with HUS we were dealing in the acute phase sera with IgM and IgG and in the follow-up and the control group we were dealing with IgG because they were all asymptomatic.

The persistence of anti-Stx2 antibodies was investigated in 49 samples from HUSrec patients at different follow-up periods. The frequency of anti-Stx2 reactive plasma in HUSrec group was similar to the HUS acute group, suggesting a specific antibody response which was long-lasting. This finding is in sharp contrast with the short persistence of the anti-Stx antibodies in Germany. In fact, Ludwig et al. [17] found that the antibody levels of 50% of the patients with HUS had decreased below the respective cut-off levels at 3 months after the onset of diarrhea. We do not know if the longer duration of the antibody response in Argentinean patients is supported by the continuous antigen re-stimulation, or if it is related to methodological differences in the used assays. However, the antibody response against the Stx2 B subunit tends to decrease faster than the antibody response against Stx2 A subunit. In fact, not only the frequency of anti-Stx2 B antibodies decreased in HUSrec patients, but also their ELISA titer decreased over time. Consequently, HUSrec group showed an increased frequency of positive samples for the A subunit compared to the NHC group (Fig. 2B) (HUSrec = 66% vs NHC = 47%), while there were not differences in the frequency of positive samples for the B subunit (HUS rec = 53% vs NHC = 53%). These findings strengthen the need for investigating individual samples sequentially.

Although the biological significance of antibodies against both Stx2 subunits is not known and requires further study, recent evidence suggests that specific antibodies for Stx2 A subunit may be important for protection [38]. In fact, monoclonal antibodies directed to the Stx2 A subunit have been proved to be as protective or more than those directed to the B subunit [39], [40]. Although the involved mechanisms are still a matter for discussion, it has been recently reported that anti-Stx2 A subunit antibodies interfere with the retrograde transport of the toxin, and may interact with Stx2 when it is still bound to membrane receptors [41]. In addition, antibodies directed to Stx2-A subunit as opposed to those directed against the B subunit, have broad-spectrum activity that includes other Stx2 variants, such as Stx2c [39], [42].

In conclusion, this is the first report describing seroepidemiological data about anti-Stx antibodies in the pediatric Argentinean population, this is in healthy and HUS-suffering children, through accurate and sensitive techniques such as ELISA and WB. This information not only could provide comprehensive immunological data as a basis for future immunization schedules to Stx, but also encourage laboratories from the National Health Surveillance System to standardize both techniques in order to evaluate a bigger population that include individuals from areas with different incidence of HUS.
